# Advances in bevacizumab in colorectal cancer: a bibliometric analysis from 2004 to 2023

**DOI:** 10.3389/fonc.2025.1552914

**Published:** 2025-03-26

**Authors:** Hao Chen, Yeqing Lei, Junjie Zhou, Chenhui Lv, Qijia Xuan

**Affiliations:** ^1^ Department of Oncology, The Fourth Affiliated Hospital, Zhejiang University School of Medicine, Yiwu, China; ^2^ Department of Gastroenterology, The Fourth Affiliated Hospital, Zhejiang University School of Medicine, Yiwu, China; ^3^ Department of Radiology, The Fourth Affiliated Hospital, Zhejiang University School of Medicine, Yiwu, China

**Keywords:** bevacizumab, colorectal cancer, citespace, VOSviewer, bibliometric analysis

## Abstract

**Background:**

Bevacizumab is a primary focus in the clinical application and research of metastatic colorectal cancer (mCRC) patients. This study aims to analyze publications on bevacizumab and CRC to explore and identify the trends and frontiers of this field.

**Methods:**

We collected 4,164 articles on bevacizumab and CRC from the Web of Science Core Collection (WoSCC). CiteSpace, VOSviewer, R-bibliometrix, and Microsoft Excel were utilized for analysis and visualization.

**Results:**

The United States, Japan, and China are the leading countries in this field. The National Cancer Institute and the University of Pisa share the top position for the highest number of publications. Personalized therapy, innovative combination treatments, mechanisms of resistance, and new drug development are enduring focal points and future research directions.

**Conclusions:**

This study provides the first bibliometric analysis of research on bevacizumab and CRC, revealing the current status and future directions of this field.

## 1 Introduction

Bevacizumab has been used for two decades as the first antiangiogenic medication in clinical practice and the first available targeted treatment for patients with advanced colorectal cancer (CRC) (1, 2). It functions by binding to vascular endothelial growth factor-A (VEGF-A) isoforms, thereby inhibiting the activation of the VEGF signaling pathway that is crucial for promoting neovascularization. Thus, bevacizumab has been primarily directed towards tumor types that are recognized as being angiogenesis-dependent, including metastatic colorectal cancer (mCRC) (2). A meta-analysis demonstrated that mCRC patients with primary tumor resection (PTR) have better survival when managed with bevacizumab (3). Yet, despite bevacizumab’s established role in the treatment strategy for mCRC, not all patients benefit from this therapy, and the prediction of bevacizumab’s efficacy in mCRC continues to be a subject of active investigation. In addition, the issue of bevacizumab resistance is a major clinical challenge due to crossover and bypass mechanisms between pathways. In addition, the resistance observed with anti-VEGF drugs implies that tumors can also promote angiogenesis and tumor progression via alternative vascular pathways. For example, research indicates that tumors resistant to anti-VEGF treatment exhibit upregulation of several angiogenic factors, including Angiopoietin-2 (Ang2), fibroblast growth factor (FGF), platelet-derived growth factor (PDGF), and placental growth factor (PlGF) (4–8). Combining VEGF targeting with the inhibition of these upregulated angiogenic factors has shown favorable outcomes in certain studies (9–11).

Although early screening and various therapeutic approaches have improved the management of CRC, significant challenges remain in treatment efficacy, patient survival, and quality of life. Key challenges involve disease heterogeneity, inter-patient variability, resistance to therapy, and the demand for innovative treatment approaches (12, 13). Among these, the efficacy and safety of bevacizumab in clinical applications remain a focus of attention, particularly regarding its effectiveness and tolerability among different patient groups (14, 15). Specialized literature on bevacizumab and colorectal cancer is emerging. However, no bibliometric studies on CRC and bevacizumab have been reported. Bibliometrics employs a spectrum of quantitative and empirical assessment tools to quantify and evaluate scholarly output within a defined academic domain. Through the integration of bibliometric analytics and visualization tools, a comprehensive and objective representation of the field’s landscape is rendered, equipping investigators with insightful visual data and indicative pathways for future investigative endeavors. This research intends to perform a systematic bibliometric analysis of the studies on CRC and bevacizumab during the period from 2004 to 2023. CiteSpace and VOSviewer are used for further visualization of the analysis results. The analysis reveals that personalized treatment will be a research direction for this subject. Current literature highlights the necessity of additional studies to comprehensively understand the resistance mechanisms of bevacizumab and to verify the efficacy of new therapies, thus optimizing the long-term use of bevacizumab and increasing treatment options for CRC.

## 2 Materials and methods

### 2.1 Data collection

The Web of Science Core Collection (WoSCC) provided the data used in this investigation. The study was strictly concentrated on publications in the English language. Additionally, only original research articles were included in our study. The search approach combined the subjects of CRC and Bevacizumab using the following search formula: [TS = (“Colorectal Neoplasm” OR “Neoplasm, Colorectal” OR “Colorectal Tumors” OR “Colorectal Tumor” OR “Tumor, Colorectal” OR “Tumors, Colorectal” OR “Neoplasms, Colorectal” OR “Colorectal Cancer” OR “Cancer, Colorectal” OR “Cancers, Colorectal” OR “Colorectal Cancers” OR “Colorectal Carcinoma” OR “Carcinoma, Colorectal” OR “Colorectal Carcinomas” OR “Carcinomas, Colorectal”)] AND TS = (Bevacizumab). The inclusion/exclusion criteria are as follows (1): The publication period was from January 1, 2004, to December 31, 2023; (2) The language was set to English only; (3) The publication type was article, excluding review, meeting abstract, book chapters, early access, correction, letter, and other document types; (4) Excluded publications not related to bevacizumab in CRC by reading abstract and full text. As of October 1, 2024, a total of 4164 original English-language articles on Bevacizumab and CRC published from 2004 to 2023 were identified. The selection process of the studies is depicted in a flowchart (**Supplementary Figure S1**).

### 2.2 Data analysis

All records of the collected papers, including title, author, country, institutions, journal, keywords, and references, were exported to a plain text file and then imported into Microsoft Excel 2016 (Microsoft, Washington, USA), CiteSpace (version 6.3 R3 Advanced), and VOS Viewer (version 1.6.20) for qualitative and quantitative analysis. Specifically, database management and annual publication analysis were performed by Microsoft Excel 2016. Reference collaboration analyses, the dual-maps overlay of journals, literature bursts, and keyword bursts were carried out with CiteSpace. The VOSviewer was utilized for analyzing co-citation and co-occurrence, as well as for conducting and visualizing the literature network map.

## 3 Results

### 3.1 Publication and citation analysis

**Figure 1:**
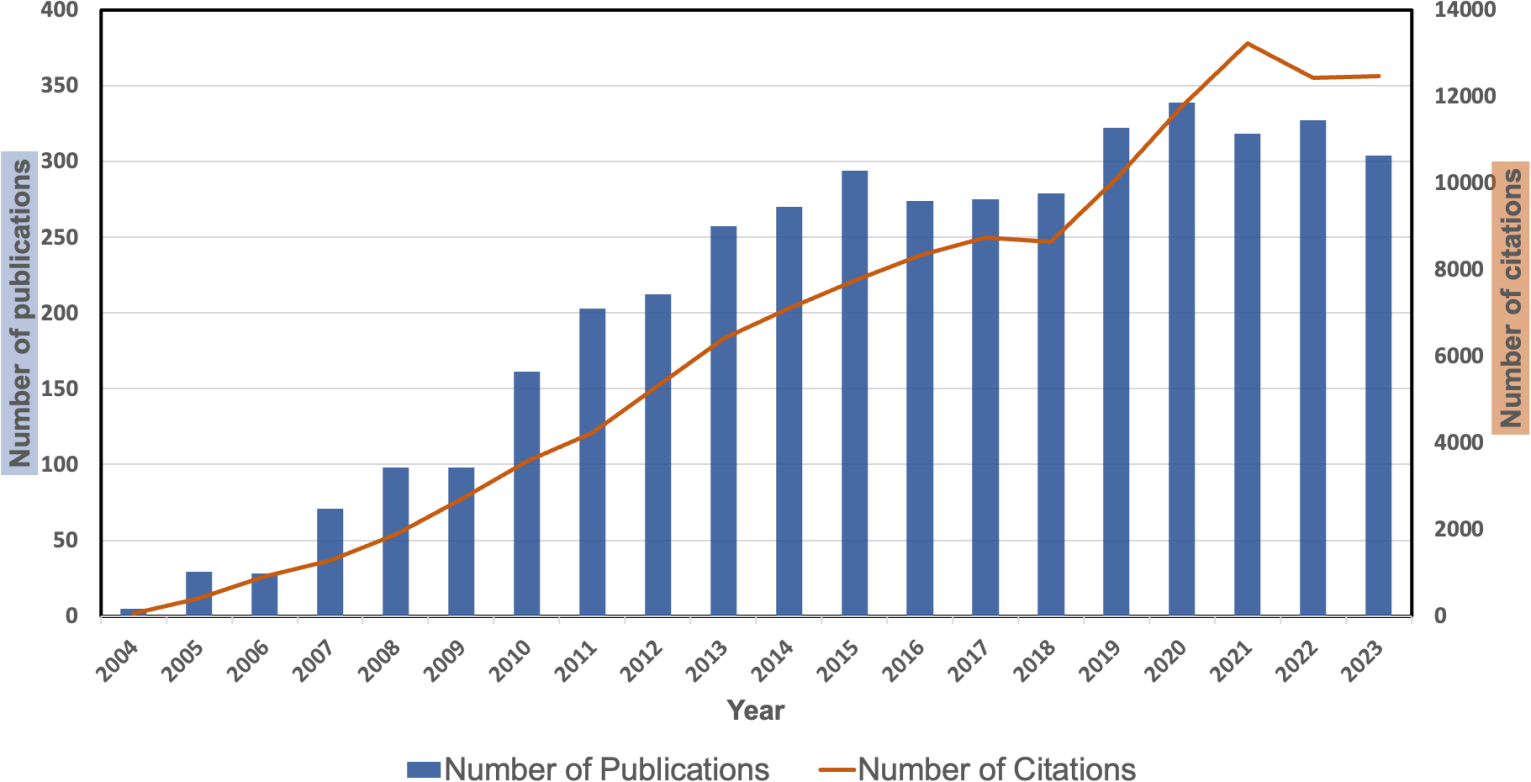
The number of articles about bevacizumab and colorectal cancer per year from 2004 to 2023.

As seen in **Supplementary Figure S1**, we retrieved 4,164 papers that focused on bevacizumab and CRC from the WoSCC database. **Figure 1** presents the annual trends in publication output and citation frequency. It shows that from 2004 to 2023, the number of publications and citations on bevacizumab and CRC research exhibited an overall upward trend. According to the peak curve of publications, it can be divided into four periods: 2004-2015, 2016-2017, 2018-2020, and 2021-2023. From 2004 to 2015, publication outputs during this period accounts for 41.5% (1726) of the total publications, and it peaks in 2015. From 2016-2017, Annual publication output remained stable, accounting for 13.2% (549) of the total. From 2018-2020, The annual output of publications shows an increasing trend, accounting for 22.6% (940) of the total. Since 2020, there was a sudden decline in the number of annual publications compared to the previous year, which may be due to the impact of the COVID-19. It is worth noting that 2022 has the highest number of publications (339), while 2023 has the highest number of citations (13232). On average, 208.2 papers were published per year, with each paper receiving 30.6 citations.

### 3.2 Analysis of countries and institutions

**Figure 2:**
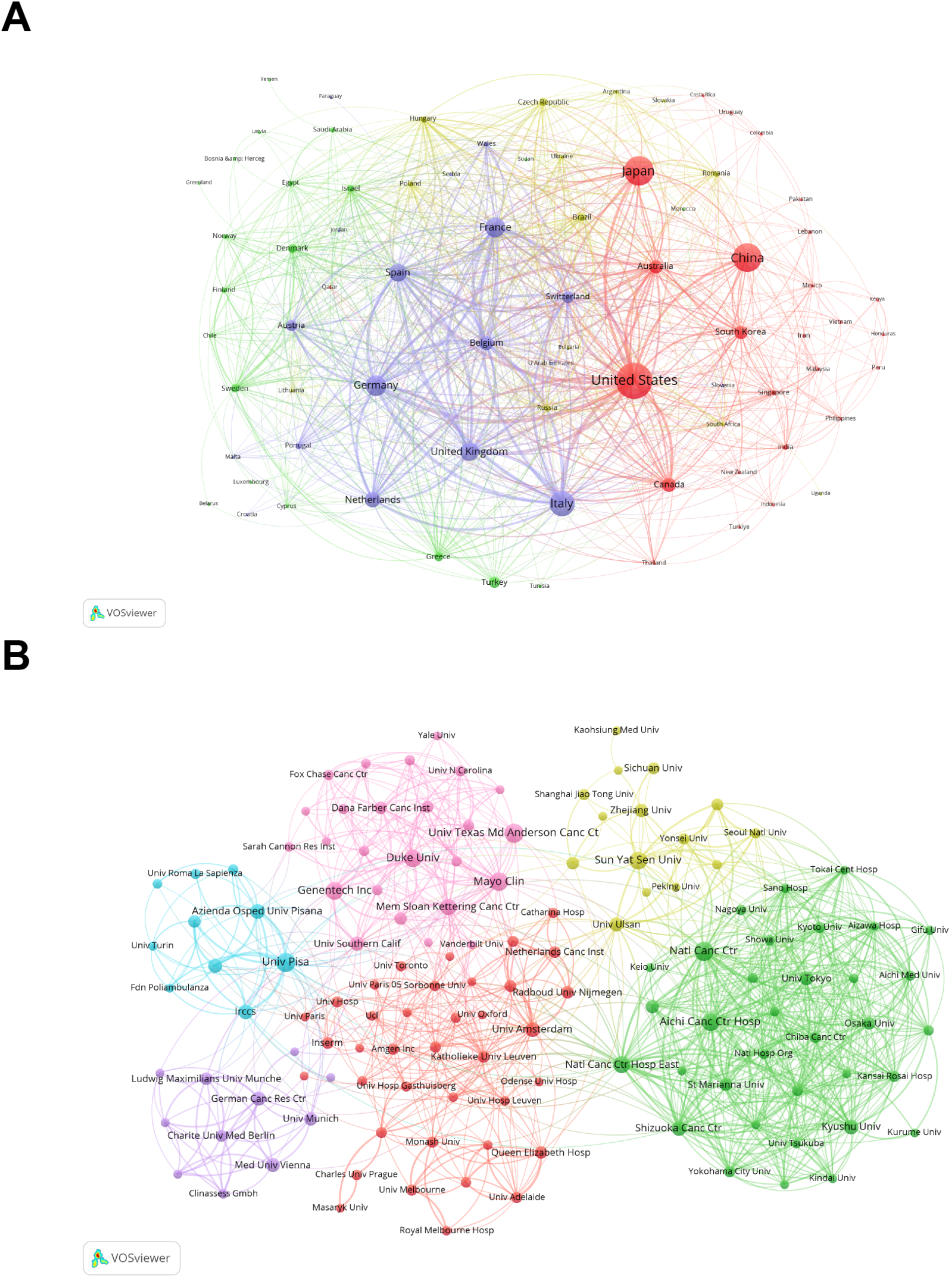
The network maps showing countries/regions **(A)** and institutions **(B)** involved in the research on bevacizumab for colorectal cancer.

**Supplementary Table S1** presents the top ten countries ranked by the number of papers, citation frequency, and total link strength. USA is the most prominent contributor in this field, ranking first in the number of publications, citations, and total link strength. **Figure 2A** divides all countries into four clusters by color. The red cluster, which mostly consists of the United States, Japan, China, and Australia, is the biggest network cluster. The blue cluster follows, including European nations like Italy, Germany, and the United Kingdom. The smaller yellow and green clusters primarily comprise the remaining European countries and a few countries from other regions. Collaboration is relatively strong between the red and blue clusters and within each cluster, with the United States showing the closest ties to other countries.

**Supplementary Table S2** shows the top 10 institutions ranked by the number of published papers and total link strength. In terms of publication volume, Univ Pisa and Natl Canc Ctr lead with 96 papers, followed by Univ Texas Md Anderson Canc Ctr (95) and Mayo Clin (87). In the total link strength rankings, the top four are Japanese institutions: Aichi Canc Ctr Hosp ranks first (401), followed by Natl Canc Ctr Hosp East (398), Natl Canc Ctr (350), and Shizuoka Canc Ctr (320). **Figure 2B** categorizes all institutions into six color-coded sections. Connections within each cluster are strong, especially in the green cluster representing Japanese institutions, though inter-cluster connections are relatively limited.

### 3.3 Author and co-cited author

**Figure 3:**
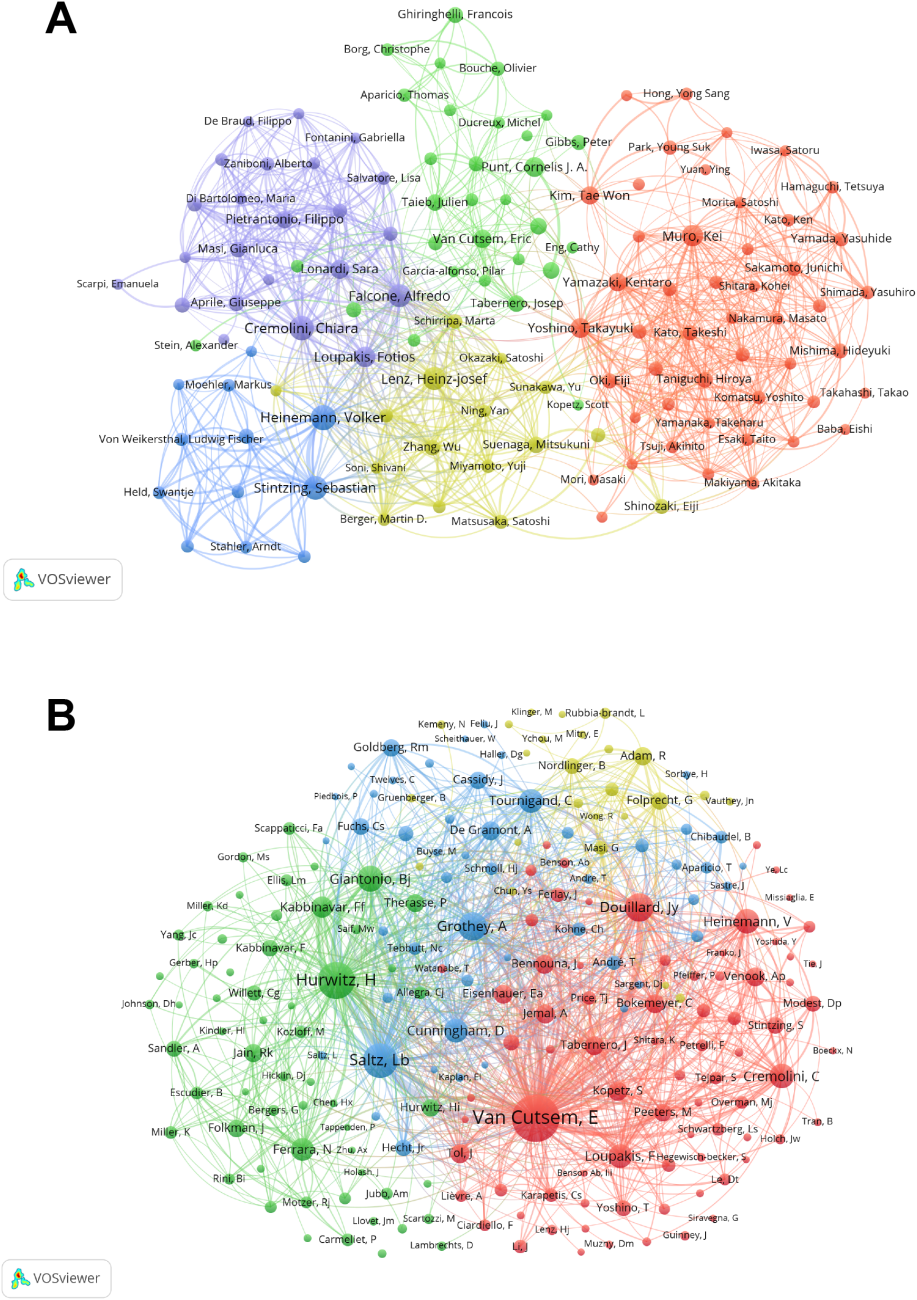
Co-author **(A)** and Co-cited-author **(B)** in the field of bevacizumab for colorectal cancer.

The top ten authors in this field are listed in **Supplementary Table S3**. Heinemann Volker is among the top ten scholars in terms of publications, followed by Cremolini Chiara, who had over 70, and the other eight writers, who had over 40. **Figure 3A** depicts a cluster analysis of co-authorship networks, indicating that collaboration in this field is relatively concentrated within teams and has not yet developed into a highly interconnected and expansive network. The connections between nodes form five close cooperation teams.

We conducted a statistical and cluster analysis of the co-citation network of authors in the domain of bevacizumab and CRC research, as presented in **Supplementary Table S3**; **Figure 3B**. Van Cutsem E emerged as the sole author in the top 10 with a co-citation count of 2500, while two authors exceeded 1000 citations and seven surpassed 600. In the total link strength rank, Van Cutsem E secured the top position with 39,156, trailed by Saltz Lb at 20,486 and Hurwitz H at 19,427. **Figure 3B** provides a visual representation of the academic collaboration network within this field. Van Cutsem E, Saltz Lb and Hurwitz H, who are the most active authors in the red, blue, and green clusters respectively.

### 3.4 Journal and co-cited journal

**Figure 4:**
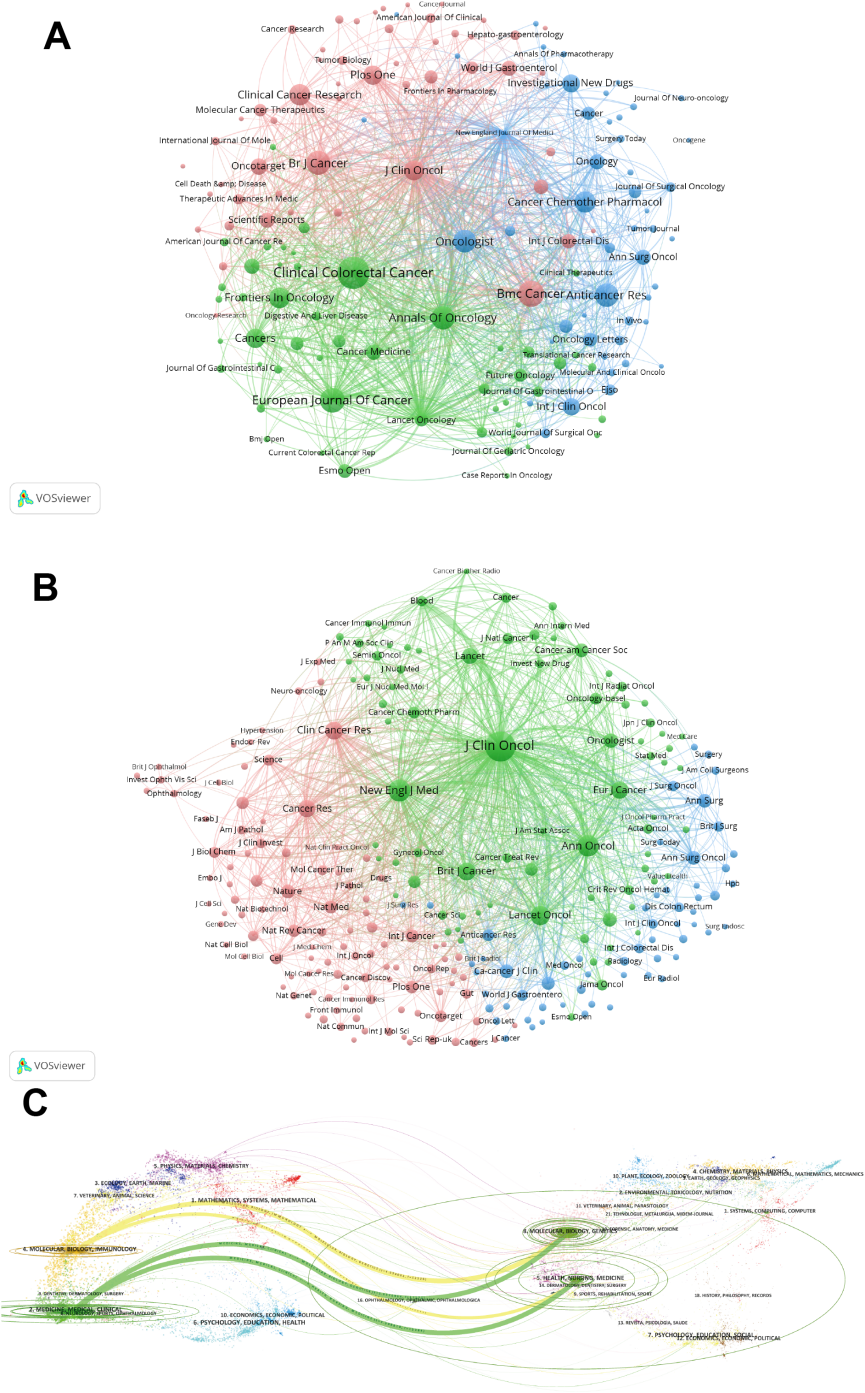
**(A)** Bevacizumab and colorectal cancer related journals. **(B)** Co-cited-journal analysis related to bevacizumab for colorectal cancer. **(C)** The dual-map overlay of journals.

**Supplementary Table S4** presents the top 10 journals and co-cited journals, detailing their publications, co-citation frequencies, impact factors (JCR2023), and their JCR quartiles. Furthermore, we employed cluster analysis to categorize all journals into three distinct groups, as depicted in **Figure 4A**. The journal network demonstrates a high degree of connectivity and extensive interlinks. **Figure 4B** depicts the cooperation among co-cited journals. J Clinical Oncology, Clinical Cancer Research, and Annals of Surgical Oncology are identified as particularly influential within the green, pink, and blue clusters, respectively.

The dual-map overlay in **Figure 4C** elucidates the topical distribution within the scientific journal landscape. The labels on the left-hand side of the dual map correspond to citing journals, while those on the right-hand side denote cited journals. The colored pathways signify the citation connections between them. **Figure 4C** highlights the two predominant citation trajectories in this domain. The most robust citation links are observed from the MEDICINE/MEDICAL/CLINICAL journals to the HEALTH/NURSING/MEDICINE journals.

### 3.5 Analysis of keywords

Keywords serve as a representation of the principal content within a scholarly article, enabling us to discern research trends and cutting-edge areas. The top 20 keywords are shown in **Supplementary Table S5**. “Bevacizumab” and “colorectal cancer” were excluded, as they constitute the search query. Chemotherapy, mCRC and fluorouracil occupied the top 3 positions for the keyword occurrences. The keyword “chemotherapy” also held the highest total link strength.

**Figure 5:**
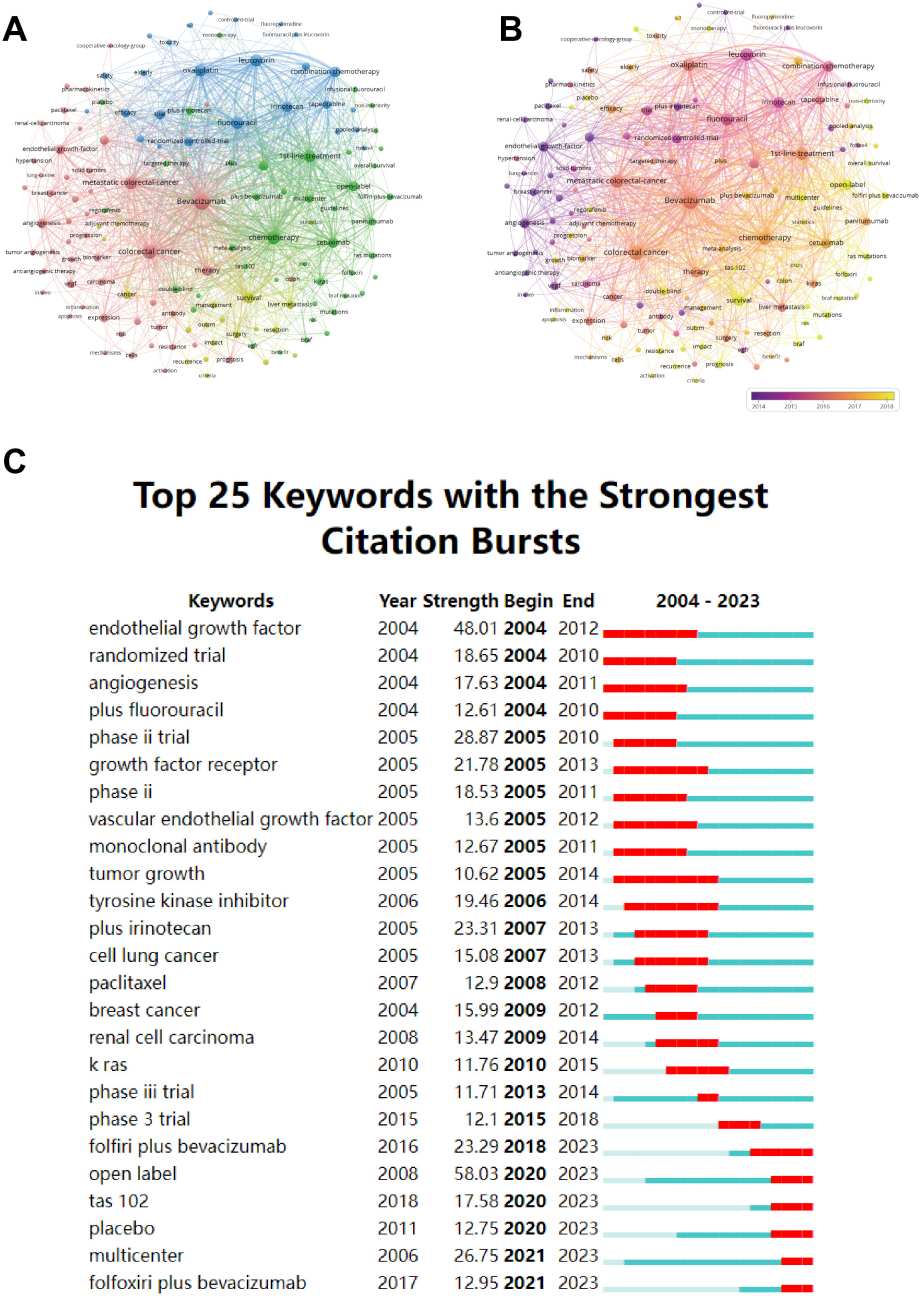
**(A)** Cluster diagram of keyword co-occurrence analysis network. **(B)** Co-occurrence network analysis graph of keywords, different colors represent different mean published years. **(C)** The twenty-five keywords with the strongest citation burst in bevacizumab for colorectal cancer.

**Figure 5A** illustrates that the keywords within the red network are related to oncogenesis and therapeutics. The yellow cluster is associated with clinical management and prognosis. The green cluster concentrates keywords related to first-line treatment. The keywords in the blue cluster are related to clinical chemotherapy regimens. **Figure 5B** adds a time dimension to **Figure 5A** to show the temporal evolution trend of the keyword clustering analysis.

Burst terms indicate keywords that emerge with high frequency during a specific period. The top 25 burst keywords are shown in **Figure 5C**. The early burst keywords include “endothelial growth factor” and “angiogenesis” which focus on the bevacizumab’s mechanism of action. The middle stage reveals a sudden surge in keywords such as “phase III trial” and “tyrosine kinase inhibitor” indicating a concentration on assessing the efficacy and safety of bevacizumab. The recent burst keywords, featuring “folfoxiri plus bevacizumab”, “tas 102” and “multicenter”, underscore the exploration of novel combinations of therapies within the larger, more extensive cohort.

### 3.6 Reference analysis

**Figure 6:**
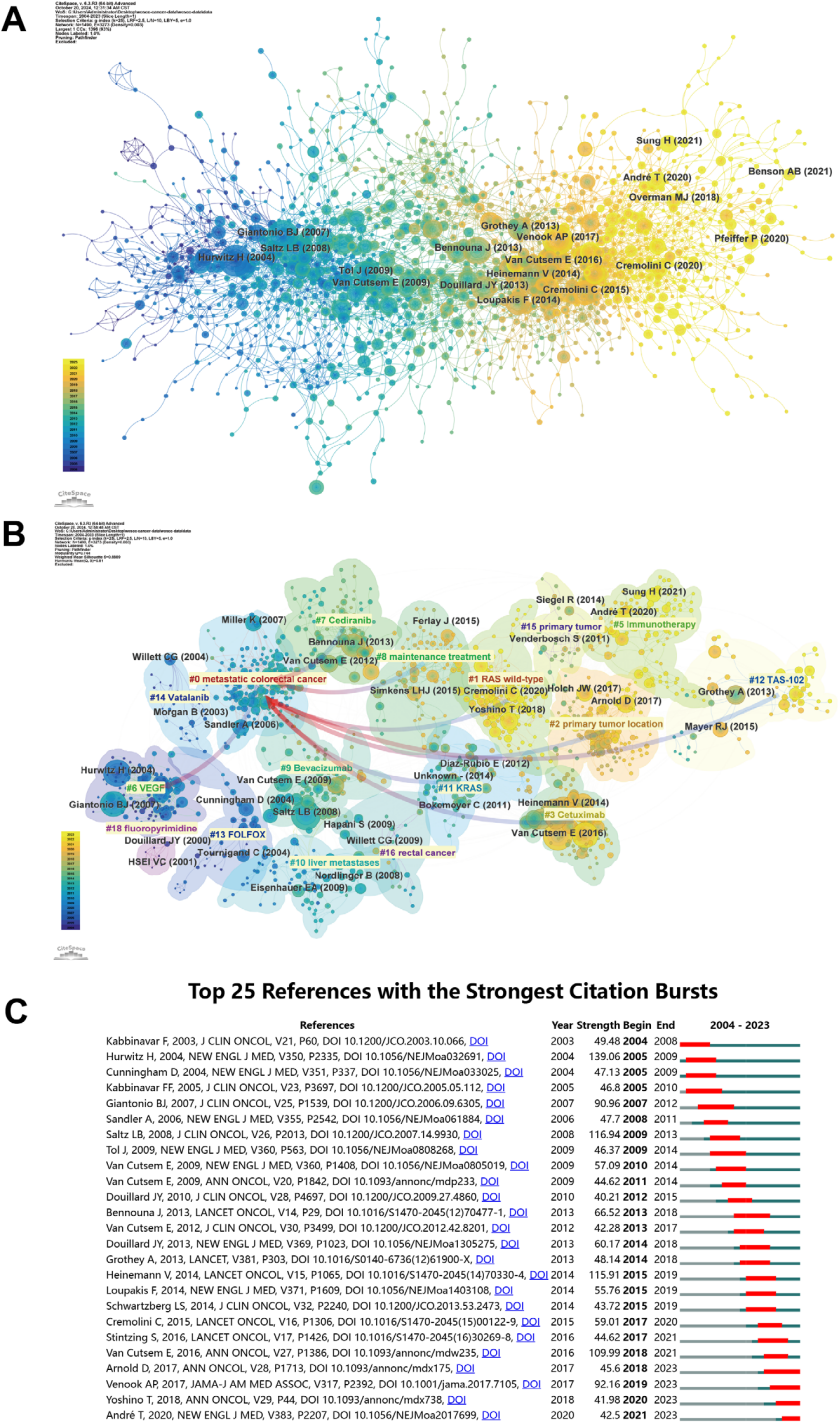
**(A)** The co-citation network of literature and **(B)** labels clustering of co-cited literature. **(C)** The twenty-five references with the strongest citation burst in bevacizumab for colorectal cancer.

**Supplementary Table S6** lists the top 10 most frequently cited references. The most frequently cited paper is “Bevacizumab plus irinotecan, fluorouracil, and leucovorin for metastatic colorectal cancer” by Hurwitz H et al., published in New England Journal of Medicine in 2004, which has been cited 8,288 times (16). **Figure 6A** displays the primary cited references. **Figure 6B** depicts the associations between these references, categorized into clusters of different topics. The largest and most notable cluster #0 metastatic colorectal cancer contains the highest number of publications. In terms of timeline, the earliest research area is #6 VEGF, which subsequently evolved into #0 metastatic colorectal cancer. Furthermore, the current research frontier focuses on #1 RAS wild-type, #2 primary tumor location, #3 Cetuximab, #7 Cediranib, #8 maintenance treatment, #11 KRAS, and #12 TAS-102. **Figure 6C** displays the 25 most influential references. The first citation burst in this field occurred in 2004, with subsequent bursts occurring annually from 2007 to 2015. In 2017, Venook AP et al. published a paper in JAMA titled ‘Effect of First-Line Chemotherapy Combined With Cetuximab or Bevacizumab on Overall Survival in Patients With KRAS Wild-Type Advanced or Metastatic Colorectal Cancer’, exhibiting significant citation burst (Strength = 92.16) from 2019 to 2013 (17). This suggests that the delineation of treatment population is becoming more nuanced and that research into personalized therapy for CRC is still ongoing.

To better present the citations of the top 10 references, we have plotted their annual citations since publication in **Supplementary Figure S2**. The size of the circles indicates the number of citations for each paper, with larger circles representing higher citation rate and greater influence. Notably, the study by Andre T et al. published since 2020 has received considerable citations. Conversely, the citations for articles by Hurwitz, H, Giantonio, BJ, Tol, J and Lee, S have declined in recent years.

## 4 Discussion

In this study, we conducted a comprehensive analysis of 4,164 papers on bevacizumab and CRC published between 2004 and 2023 using bibliometric methods, along with data visualization. Our analysis combined quantitative and qualitative methods, depicting the evolution of the field and its future trends across various dimensions such as annual publication volume, journals, authors, institutions, keywords, and references. Our comprehensive research results will be explored in the upcoming sections, providing deep insights from different viewpoints.

### 4.1 Publication and citation trends

Based on our analysis, the volume of publications and citations regarding bevacizumab in CRC has steadily risen in recent years, surpassing 300 publications annually and accumulating over 12,000 citations. In 2004, a landmark trial on anti-angiogenic therapy for CRC was officially launched, including Phase II and III AVF2107 trials, which confirmed that chemotherapy (irinotecan, 5-FU, and leucovorin) combined with bevacizumab is superior to chemotherapy combined with placebo, achieving significant results that sparked further research interest (16). Consequently, the publication volume in this area had increased year by year, with the mechanisms of bevacizumab, its clinical applications in CRC, and efficacy assessments becoming research hotspots in recent years. At the same time, it was the most cited document in total, with 8,288 citations, well ahead of the second and third places with 1,863 and 1,230 citations, respectively.

### 4.2 National/institutional publishing trends and cooperation

The United States, Japan, and China are the main contributors in this field. The United States leads the world in the number of publications, with 1,022 papers, and it also has the highest citation frequency and the broadest collaborations with other countries or regions. The most high-profile study was the landmark CRC anti-angiogenesis therapy trial in 2004, which demonstrated the significance of adding bevacizumab to combination chemotherapy to improve survival in patients with mCRC (16). The dominance of the red cluster (United States, Japan and China) in terms of publications (57.1% of total output) reflects the far-reaching research dynamics in a given geographical region. There are two main drivers of this phenomenon: 1. burden of disease: according to global cancer statistics, North America and East Asia rank high in age-standardised incidence rates for both colon and rectal cancer (18); 2. Research: The Red Cluster countries are global leaders in science and technology and research investment, which drives continued research into CRC. The distribution of institutions largely corresponds to the countries. **Supplementary Table S2** indicates that five of the ten institutions with the highest publication volumes are from the United States. The National Cancer Institute and the University of Pisa rank jointly as the institutions with the highest number of publications within the collaborative network. Aichi Cancer Center Hospital is the institution that collaborates most extensively with other institutions. Genentech has the highest citation frequency, likely due to the generous political and funding support offered by the United States to scholars conducting in-depth research in this field.

### 4.3 Leading authors in the field

Among the authors, the top ten each published at least forty papers, with Professor Heinemann Volker leading with seventy-five papers. Eric Van Cutsem ranks first among co-cited authors and is also the scholar with the broadest collaborations with other authors. The works of the ten most co-cited authors have received at least 640 citations, highlighting their outstanding impact and significant contributions regarding bevacizumab and CRC. The bevacizumab extended access trial (BEAT) involving Eric Van Cutsem confirmed that bevacizumab can be combined with any chemotherapy regimen commonly used in clinical practice (19). Heinemann Volker has not only published a large number of articles but also has a high citation frequency. In the FIRE-3 randomized clinical trial he led in 2020, it was found that among patients with left-sided tumors, folinic acid, 5-fluorouracil, and irinotecan (FOLFIRI) combined with cetuximab significantly increased objective response rate (ORR) and extended overall survival (OS) compared to FOLFIRI combined with bevacizumab (20). As key figures in these areas continue to bring forth new research findings, the field is continuously advancing.

### 4.4 Journal publications, citations and impact

The largest number of publications was Clinical Colorectal Cancer (IF=3.3, Q2) with 187 publications. Journal of Clinical Oncology ranks first, with an impact factor of 42.1 and citation times up to 21443. It is noteworthy that five of the top ten journals are positioned in Q1 of the JCR, and four of these ten journals possess an impact factor exceeding 5, which is evidence of the high impact of journals in this field.

### 4.5 Extracting hotspots and frontiers from keywords and reference analysis

Keywords and references analysis can help identify research hotspots and frontiers, unveil the relationships and hierarchies among different research themes, and monitor research dynamics, thereby aiding in understanding current research trends and future directions. Scholars have been researching the application of bevacizumab in CRC treatment since 2004, exploring multiple directions such as beneficiary population screening, combination therapies, and novel drug development, with continued interest in those reference clusters shown in **Figure 6B** to the present. A comprehensive analysis of the development of references and keywords over the last two decades suggests that the focus in this area is likely to be on personalized patient treatment, novel therapeutic combinations, and mechanisms of resistance in long-term therapy.

#### 4.5.1 Precision and personalized treatment

The literature burst analysis suggests that precision and personalized treatment is a current significant research objective. Identifying specific genetic alterations can guide the development of targeted therapies, enhancing the feasibility of personalized treatments. Emerging research underscores the importance of genetic and molecular markers in predicting bevacizumab efficacy. For example, Yang J et al. developed a gene pair signature (GPS) based on the primary site to predict the response and prognosis of mCRC to bevacizumab. Results show that the clinical outcomes of the predicted response group were significantly better than those of the non-response group, indicating that the 64-GPS is an objective and reliable genetic feature for predicting mCRC patients’ response to bevacizumab, effectively assisting clinical treatment decisions (21). Similarly, Zuurbier et al. identified apelin (APLN) as a key biomarker through DNA microarray analysis, quantitative real-time polymerase chain reaction (qRT-PCR), and immunohistochemistry. Elevated APLN levels were associated with poor progression-free survival (PFS) and suboptimal response to bevacizumab in CRC patients (22). These insights emphasize the potential of integrating molecular diagnostics into clinical workflows to identify patient subgroups most likely to benefit from bevacizumab. The adoption of molecular markers such as GPS and APLN represents a critical step toward precision oncology. These biomarkers allow for stratifying patients based on bevacizumab sensitivity, thereby reducing unnecessary treatments and enhancing clinical outcomes. Future research should prioritize validating these markers in diverse patient cohorts and exploring their integration into routine diagnostic pipelines.

#### 4.5.2 Novel combination therapies

Combination therapies involving bevacizumab have demonstrated significant potential in enhancing outcomes for mCRC patients. While standard first-line treatments commonly combine bevacizumab with fluoropyrimidine-based chemotherapy, clinical benefits remain constrained, necessitating further exploration of novel combinations. As shown in **Figure 5C**, the recent burst keywords, including “tas 102”, “folfoxiri plus bevacizumab”, and “multicenter”, indicate the continued interest of researchers in new combination therapies. In the CAIRO2 Phase III trial, researchers evaluated adding an anti-EGFR agent, cetuximab, to the XELOX chemotherapy regimen (capecitabine, oxaliplatin) combined with bevacizumab for first-line treatment in mCRC. The results showed no improvement in OS or response rates, suggesting a bottleneck in treatment for these patients (23). Emerging combinations with bevacizumab have demonstrated promise in overcoming resistance to conventional chemotherapy. TAS-102, which combines trifluridine and tipiracil hydrochloride, is a novel oral chemotherapy drug approved for mCRC treatment. Its unique mechanism of action and metabolism have shown efficacy in 5-FU-refractory patients, playing an important role in advanced mCRC treatment (24). For combination therapy, a Phase II trial found TAS-102 combined with bevacizumab led to longer PFS than TAS-102 alone (25). The SUNLIGHT phase III clinical trial revealed that combining TAS-102 (trifluridine/tipiracil) with bevacizumab significantly improved OS (10.8 vs. 7.5 months) and PFS (5.6 vs. 2.4 months) compared to TAS-102 monotherapy in refractory mCRC patients (26). Large-scale randomized controlled studies are needed to further evaluate the efficacy and safety of TAS-102 combined with anti-angiogenic tyrosine kinase inhibitors (TKIs) (such as fruquintinib or regorafenib). In a meta-analysis by Cremolini C et al., FOLFOXIRI (fluorouracil, leucovorin, oxaliplatin, and irinotecan) plus bevacizumab was shown to enhance OS, PFS, ORR, and R0 resection rates in mCRC patients compared to doublet chemotherapy with bevacizumab, albeit with moderately increased toxicity (27). These findings reinforce the need for personalized decision-making when incorporating bevacizumab into chemotherapy regimens. As immune checkpoint inhibitors (ICIs) have achieved success in melanoma, renal cell carcinoma, and NSCLC, researchers are now exploring their use in CRC. Results indicated that only mCRC patients with mismatch repair deficient (dMMR)/microsatellite instability-high (MSI-H) benefit from this treatment, and these patients account for only 5% of mCRC cases (28). Pan QZ et al. conducted a Phase III trial showing that adding adoptive cell immunotherapy to first-line XELOX plus bevacizumab significantly improved PFS and OS in previously untreated mCRC patients (29). The ASTRUM-015 phase II/III clinical study evaluated the efficacy and safety of serplulimab versus placebo combined with bevacizumab and XELOX chemotherapy for first-line treatment of mCRC patients. The latest follow-up at 24.4 months showed that serplulimab combined with bevacizumab and chemotherapy significantly improved PFS in MMR-proficient (pMMR)/microsatellite stable (MSS) mCRC patients, providing crucial evidence-based support for these so-called “cold tumors” in immunotherapy (30).

While combination therapies can improve patient survival, there are corresponding adverse events. In the CAIRO2 phase III clinical trial, the addition of cetuximab to XELOX chemotherapy in combination with bevacizumab resulted in more grade 3 or 4 adverse events (81.7% vs 73.2%, P=0.006) that were attributed to cetuximab-associated cutaneous adverse reactions, as well as a decrease in patients’ quality of life (P=0.03) (23). In the phase II trial conducted by Pfeiffer P et al, TAS-102 combined with bevacizumab demonstrated more neutropenia compared to TAS-102 alone (67% vs 38%) (25). Similarly in the SUNLIGHT phase III trial, TAS-102 in combination with bevacizumab showed a higher incidence of severe neutropenia (43.1% vs 32.1%) compared to TAS-102 alone, possibly related to increased accumulation of phosphorylated trifluridine promoted by bevacizumab. However, the addition of bevacizumab to TAS-102 did not increase the risk of serious adverse events or adverse events leading to treatment interruption (26). Compared to two-agent chemotherapy combined with bevacizumab, FOLFOXIRI combined with bevacizumab was associated with a higher incidence of neutropenia (45.8% vs. 21.5%), febrile neutropenia (6.3% vs. 3.7%), nausea (5.5% vs. 3.0%), mucositis (5.1% vs. 2.9%), and diarrhea (17.8% vs. 8.4). However, there was no significant increase in toxicity-related mortality (27). Serplulimab in combination with bevacizumab and XELOX chemotherapy was associated with a higher incidence of Grade 3 or higher treatment-related adverse events in the ASTRUM-015 Phase II/III study (70.9% vs. 59.6%) compared to the placebo group (30). These results suggest that while combination therapy offers significant advantages in terms of survival, it is also associated with more common side effects that need to be controlled. These toxicities are acceptable as long as overall the benefits outweigh the harms and there are no serious adverse events or adverse events leading to treatment interruption.

#### 4.5.3 Resistance mechanisms in maintenance treatment

According to **Figure 6B**, #8 maintenance treatment is one of current reference clusters. Based on existing research findings, bevacizumab combined with fluoropyrimidine drugs (like capecitabine) or used as a monotherapy in maintenance treatment has demonstrated favorable efficacy (31). Although bevacizumab enhances the prognosis of mCRC, the majority of patients exhibit intrinsic or acquired resistance to the treatment, which ultimately restricts its long-term effectiveness (32). The main mechanisms of resistance include changes in the tumor microenvironment, intrinsic adaptability of tumor cells, and compensatory activation of other angiogenesis alternative pathways. Zheng Y et al. found that the stiffness of the extracellular matrix (ECM) in the TME promotes the resistance of tumor cells by regulating lipid metabolism and cell signaling pathways (33). The inherent adaptability of tumor cells is also crucial in the resistance to bevacizumab. Qin X et al. discovered that colorectal cancer stem cells (CCSCs) mediate resistance to bevacizumab via the IL-22-STAT3 signaling pathway, suggesting that tumor stem cells may be a significant source of resistance (34). Numerous studies have indicated that factors or pathways including PlGF, Ang-2, FGF/FGFR, mesenchymal–epithelial transition factor (c-MET), transforming growth factor-β (TGF-β), interleukin (IL)-1, macrophage migration inhibitory factor (MIF), and PDGFR are upregulated, downregulated, overexpressed, or compensatorily activated in CRC patients resistant to anti-angiogenic therapy, suggesting that these factors or pathways are key to overcoming resistance to such treatments (4, 6, 35–40). Nevertheless, the research on modulators targeting these factors is still quite limited or is currently in the early stages of clinical translational studies. Targeting the mechanisms underlying resistance has become a focal point in anti-angiogenic therapy research. Qi M et al. found in a model of CRC liver metastasis that targeting FAPα-expressing hepatic stellate cells can effectively overcome anti-angiogenic resistance (41). Rigamonti N et al. found in preclinical studies that concurrently targeting VEGF and Ang-2 aids in managing resistance to VEGF-targeted therapies (9).

This study provides three clinical practice recommendations: 1. Biomarker-guided treatment personalization: Clinicians should prioritize molecular profiling (e.g., GPS signature, APLN levels) before initiating bevacizumab; 2. Sequencing of novel combination therapies: For refractory mCRC, the SUNLIGHT trial data support using TAS-102 plus bevacizumab as third-line therapy, while FOLFOXIRI plus bevacizumab remains preferred for first-line intensive treatment; 3. Drug resistance monitoring and management: Serial plasma biomarker monitoring (including Ang-2 and PlGF) enables early detection of therapeutic resistance and facilitates adaptive treatment modifications.

#### 4.5.4 Tyrosine kinase inhibitor

In addition, we found from the keywords burst analysis that tyrosine kinase inhibitor has also received significant attention. Regorafenib is the first multi-target TKI to show survival benefits in mCRC, targeting VEGFR and BRAF. In a phase III clinical trial for refractory mCRC patients, regorafenib monotherapy achieved better OS (6.4 vs 5.0 months) and PFS (1.9 vs 1.7 months) than the placebo group (42), a result later validated in an Asian population (43). In recent years, as our understanding of CRC has advanced, new drugs have emerged continuously. The randomized clinical trial FRESCO evaluated fruquintinib, a highly selective small-molecule inhibitor of VEGFR1, VEGFR2, and VEGFR3, as third-line or later treatment for mCRC in the Chinese population (44). The results showed that fruquintinib monotherapy significantly prolonged OS (9.3 vs. 6.6 months) and PFS (3.7 vs. 1.8 months) compared to the placebo group, leading to its approval by the National Medical Products Administration (NMPA) in China. Anlotinib is a novel multi-target TKI targeting receptors including VEGFR1-3 and fibroblast growth factor receptor (FGFR) 1-4. An unpublished phase II clinical study showed favorable and encouraging results for anlotinib in refractory mCRC, with a good ORR, disease control rate (DCR), and patient tolerance. A multicenter, double-blind, placebo-controlled, randomized phase III clinical trial led by Chi Y et al. evaluated the efficacy and safety of anlotinib monotherapy in refractory mCRC patients. Although the difference in OS between groups was not statistically significant, anlotinib monotherapy significantly improved PFS (4.1 vs. 1.5 months), reducing the risk of disease progression by 66% (45).

Overall, on one hand, we need to enhance the application effectiveness of bevacizumab in the treatment of CRC through ongoing clinical research and feedback from clinical practice. On the other hand, we must integrate with continuously evolving scientific research to develop more personalized treatments and novel combination therapies, overcome resistance issues, and promote the development of new drugs, aiming for longer survival, fewer adverse reactions, and higher quality of life.

### 4.6 Current status and future perspectives

Our bibliometric analysis reveals clinical realities and critical avenues for advancing bevacizumab applications in CRC.

#### 4.6.1 Current clinical landscape

1. Bevacizumab remains the first-line standard of care for mCRC, albeit with variable response rates. 2. Predictive biomarkers (e.g. APLN, GPS) are emerging but lack clinical validation. 3. Mechanisms of resistance are multifactorial (ECM remodeling, CCSC plasticity, angiogenic redundancy). 4. New drugs (e.g., multi-targeted TKIs) and clinical trials are emerging to provide more treatment options for refractory mCRC patients.

#### 4.6.2 Future perspectives

Biomarker validation and implementation: While preliminary studies have identified promising predictive signatures like the 64-GPS and APLN expression patterns, their clinical utility remains constrained by limited validation across diverse ethnic populations and treatment settings. Large-scale prospective trials incorporating liquid biopsy approaches are urgently needed to establish standardized biomarker panels. Recent work by Vidal et al. demonstrates the feasibility of ctDNA-guided screening of CRC, providing a methodological framework for such validation efforts (46).

Rational combination strategies: Building on the observed keyword bursts around “folfoxiri plus bevacizumab” and “tas 102”, future studies should systematically evaluate the optimal sequencing of bevacizumab with emerging agents and synergistic combinations with immune checkpoint inhibitors in MSS/pMMR populations.

Resistance reversal: Firstly, comprehensive genomic and molecular studies are needed to reveal the exact mechanisms behind resistance; secondly, design and clinical evaluation of inhibitors targeting the multiple pathways involved in angiogenesis and resistance; and lastly, the identification and validation of biomarkers that can predict a patient’s susceptibility to resistance and guide personalized therapeutic approaches. By addressing these challenges, future therapies have the potential to delay drug resistance and expand treatment options for patients with refractory mCRC.

Survival trend: On the one hand, researchers are constantly exploring the use of bevacizumab in combination with other drugs (especially immunotherapy and novel chemotherapy drugs) in the treatment of CRC. On the other hand, a variety of new drugs and clinical trials continue to emerge. We believe that with the promotion of these factors, the survival rate and quality of life of CRC patients will continue to improve.

### 4.7 Limitations

Our study is the first bibliometric analysis focusing on the application of bevacizumab in CRC from 2004 to 2023. However, our study has inevitable limitations. Firstly, our exclusive reliance on the WoSCC database may introduce selection bias. While WoSCC is a gold-standard repository for citation indexing, it underrepresents clinical trial registries (e.g., ClinicalTrials.gov), non-English journals, and regional databases (e.g., CNKI for Chinese literature). This could disproportionately exclude contributions from developing countries, potentially skewing institutional/country rankings toward Western research ecosystems (47). Secondly, geographic disparities in scientific infrastructure and funding may explain the dominance of the US, Japan, and China in publication outputs. For instance, African and South American studies accounted for <1% of publications, reflecting systemic inequities in global cancer research participation. Thirdly, variations in publication years make it difficult to compare citations from recently published papers with those from earlier ones. **Supplementary Table S6** presents the top 10 most cited references on bevacizumab and CRC. However, the substantial publication time interval of these articles makes a direct comparison of citation frequencies unreliable and biased. Therefore, we have plotted the annual citation of the top 10 most frequently cited references since their publication, as shown in **Supplementary Figure S2**. Fourthly, the data in our study may be inconsistent due to various factors, such as institutions using different names over time. These biases align with patterns observed in bibliometric studies of other oncologic therapies.

## 5 Conclusion

In this research, we retrieved 4,164 original articles related to bevacizumab and CRC from the WoSCC database covering the period from 2004 to 2023. This study also marks the first use of VOSviewer and CiteSpace for analysis and visualization of results in this field. Our results show that the United States, Japan, and China are the top contributors to this field over the past two decades. The National Cancer Institute and the University of Pisa share the top position for the highest number of publications. Clinical Colorectal Cancer and the Journal of Clinical Oncology are the two most influential journals in this field. Eric Van Cutsem is an authoritative and significant scholar in this field. Personalized therapy, innovative combination treatments, mechanisms of resistance, and new drug development are enduring focal points and future research directions in this area. In summary, our research clarifies numerous important data in this field and reveals future research trends and frontiers. We hope this research can provide readers with a more objective and thorough insight into the domain.

## Data Availability

The datasets presented in this study can be found in online repositories. The names of the repository/repositories and accession number(s) can be found in the article/**Supplementary Material**.
