# Supplementary material for: Advances in bevacizumab in colorectal cancer: a bibliometric analysis from 2004 to 2023
Source: Front Oncol. 2025 Mar 26;15:1552914. doi: 10.3389/fonc.2025.1552914 (PMC11979164; doi:10.3389/fonc.2025.1552914)
Supplement: Supplementary file 1 [file DataSheet1.docx]

Supplementary Material

# Supplementary Figures and Tables

## Supplementary Figures

**
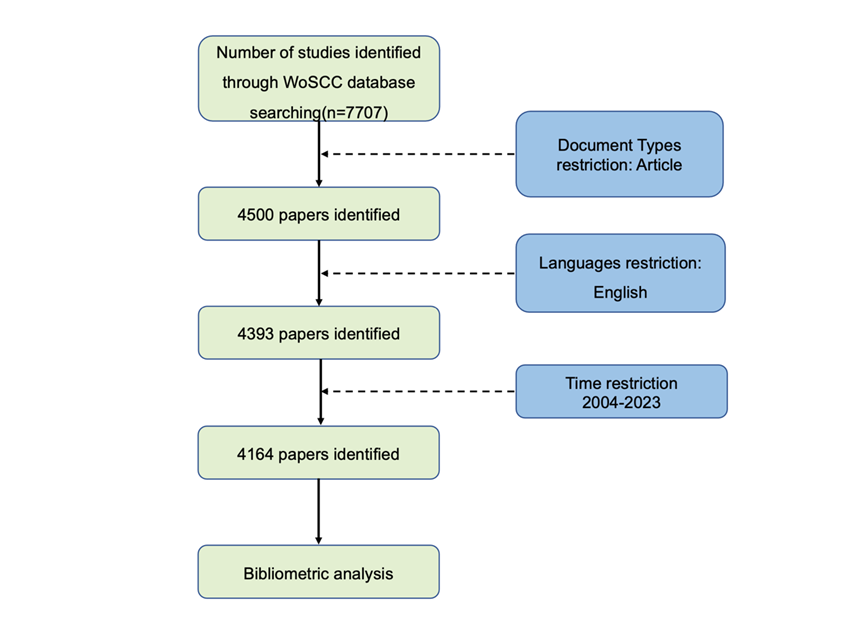
**

**Figure S1.** Flow chart of literature screening. WoSCC, Web of Science Core Collection.

**
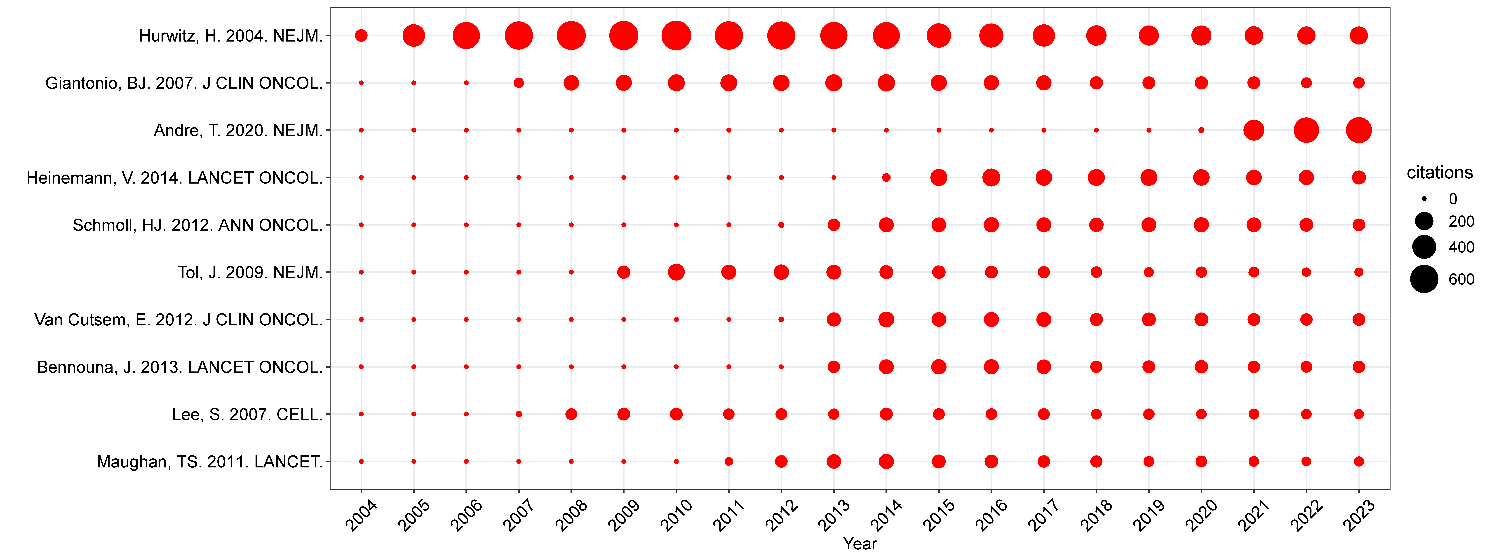
**

**Figure S2.** Annual citations of top10 highly cited references.

## Supplementary Tables

**Table S1.** Top 10 most productive countries regarding Bevacizumab and Colorectal Cancer from 2004 to 2023.

| Rank | Country | Documents | Rank | Country | Citations | Rank | Country | Total Link Strength |
| --- | --- | --- | --- | --- | --- | --- | --- | --- |
| 1 | United States | 1022 | 1 | United States | 67146 | 1 | United States | 1145 |
| 2 | Japan | 697 | 2 | Italy | 22008 | 2 | Germany | 843 |
| 3 | China | 657 | 3 | United Kingdom | 20650 | 3 | Italy | 807 |
| 4 | Italy | 511 | 4 | Germany | 19688 | 4 | Spain | 805 |
| 5 | Germany | 356 | 5 | France | 17869 | 5 | United Kingdom | 800 |
| 6 | France | 327 | 6 | Spain | 16949 | 6 | Belgium | 789 |
| 7 | United Kingdom | 281 | 7 | Belgium | 15940 | 7 | France | 786 |
| 8 | Spain | 249 | 8 | China | 13115 | 8 | Australia | 501 |
| 9 | Netherlands | 201 | 9 | Japan | 12792 | 9 | Netherlands | 448 |
| 10 | Canada | 167 | 10 | Netherlands | 12605 | 10 | Switzerland | 431 |

**Table S2.** Top 10 institutions in terms of number of articles issued and intensity of association.

| Rank | Institution | Documents | Original Country | Rank | Institution | Total Link Strength | Original Country |
| --- | --- | --- | --- | --- | --- | --- | --- |
| 1 | Natl Canc Ctr | 96 | Japan | 1 | Aichi Canc Ctr Hosp | 401 | Japan |
| 2 | Univ Pisa | 96 | Italy | 2 | Natl Canc Ctr Hosp East | 398 | Japan |
| 3 | Univ Texas Md Anderson Canc Ctr | 95 | USA | 3 | Natl Canc Ctr | 350 | Japan |
| 4 | Mayo Clin | 87 | USA | 4 | Shizuoka Canc Ctr | 320 | Japan |
| 5 | Aichi Canc Ctr Hosp | 85 | Japan | 5 | Univ Pisa | 232 | Italy |
| 6 | Genentech Inc | 84 | USA | 6 | Kyushu Univ | 230 | Japan |
| 7 | Sun Yat Sen Univ | 84 | China | 7 | Univ Tokyo | 218 | Japan |
| 8 | Duke Univ | 79 | USA | 8 | Mayo Clin | 214 | USA |
| 9 | Natl Canc Ctr Hosp East | 75 | Japan | 9 | St Marianna Univ | 201 | Japan |
| 10 | Mem Sloan Kettering Canc Ctr | 72 | USA | 10 | Hokkaido Univ Hosp | 201 | Japan |

**Table S3.** Top 10 most productive author and co-cited authors regarding Bevacizumab and Colorectal Cancer.

| Rank | Author | Documents | Country | Author | Co-citations | Country | Author | Total Link Strength | Country |
| --- | --- | --- | --- | --- | --- | --- | --- | --- | --- |
| 1 | Heinemann, Volker | 75 | Germany | Van Cutsem, E | 2540 | Belgium | Van Cutsem, E | 39156 | Belgium |
| 2 | Cremolini, Chiara | 73 | Italy | Hurwitz, H | 1596 | USA | Saltz, Lb | 20486 | USA |
| 3 | Stintzing, Sebastian | 66 | Germany | Saltz, Lb | 1378 | USA | Hurwitz, H | 19427 | USA |
| 4 | Falcone, Alfredo | 62 | Italy | Douillard, Jy | 944 | France | Douillard, Jy | 15826 | France |
| 5 | Lenz, Heinz-josef | 62 | Germany | Grothey, A | 938 | USA | Grothey, A | 14970 | USA |
| 6 | Muro, Kei | 56 | Japan | Giantonio, Bj | 792 | USA | Giantonio, Bj | 11463 | USA |
| 7 | Loupakis, Fotios | 53 | Italy | Cremolini, C | 698 | Italy | Cunningham, D | 10912 | USA |
| 8 | Punt, Cornelis J. A. | 47 | Netherlands | Heinemann, V | 693 | Germany | Tournigand, C | 10246 | France |
| 9 | Yoshino, Takayuki | 47 | Japan | Cunningham, D | 644 | USA | Cremolini, C | 9662 | Italy |
| 10 | Lonardi, Sara | 45 | Italy | Loupakis, F | 640 | ITALY | Loupakis, F | 9378 | Italy |

**Table S4.** Top 10 journals and co-cited journals regarding Bevacizumab and Colorectal Cancer from 2004 to 2023

| Rank | Journal | Publications | IF  (JCR2023) | JCR quartile | Co-cited-journal | Citations | IF  (JCR2023) | JCR quartile |
| --- | --- | --- | --- | --- | --- | --- | --- | --- |
| 1 | Clinical Colorectal Cancer | 187 | 3.3 | Q2 | J Clin Oncol | 21443 | 42.1 | Q1 |
| 2 | Bmc Cancer | 120 | 3.4 | Q2 | New Engl J Med | 7278 | 96.2 | Q1 |
| 3 | Eur J Cancer | 111 | 7.6 | Q1 | Ann Oncol | 6545 | 56.7 | Q1 |
| 4 | Br J Cancer | 103 | 6.4 | Q1 | Lancet Oncol | 4659 | 41.6 | Q1 |
| 5 | Anticancer Res | 99 | 1.6 | Q4 | Clin Cancer Res | 3554 | 10.4 | Q1 |
| 6 | Ann Oncol | 97 | 56.7 | Q1 | Brit J Cancer | 3169 | 6.4 | Q1 |
| 7 | Oncologist | 93 | 4.8 | Q1 | Eur J Cancer | 3060 | 7.6 | Q1 |
| 8 | Clin Cancer Res | 84 | 10.4 | Q1 | Cancer Res | 2860 | 12.5 | Q1 |
| 9 | Cancer Chemother Pharmacol | 83 | 2.7 | Q2 | Lancet | 2281 | 98.4 | Q1 |
| 10 | Frontiers In Oncology | 77 | 3.5 | Q2 | Oncologist | 1724 | 4.8 | Q1 |

**Table S5.** Top 20 keywords regarding Bevacizumab and Colorectal Cancer from 2004 to 2023.

| Rank | Keyword | Occurrences | Total link strength | Rank | Keyword | Occurrences | Total link strength |
| --- | --- | --- | --- | --- | --- | --- | --- |
| 1 | Bevacizumab | 2730 | 18889 | 11 | cetuximab | 712 | 5719 |
| 2 | colorectal cancer | 1929 | 13084 | 12 | irinotecan | 700 | 5886 |
| 3 | chemotherapy | 1502 | 11194 | 13 | therapy | 655 | 4832 |
| 4 | metastatic colorectal-cancer | 1341 | 9458 | 14 | open-label | 525 | 4048 |
| 5 | fluorouracil | 1178 | 9770 | 15 | phase-iii trial | 459 | 3726 |
| 6 | oxaliplatin | 1105 | 9107 | 16 | endothelial growth-factor | 453 | 3024 |
| 7 | 1st-line treatment | 1060 | 8603 | 17 | randomized controlled-trial | 446 | 3559 |
| 8 | leucovorin | 1023 | 8620 | 18 | trial | 442 | 3413 |
| 9 | survival | 797 | 5641 | 19 | angiogenesis | 433 | 2927 |
| 10 | combination chemotherapy | 716 | 5931 | 20 | phase-ii trial | 406 | 3051 |

**Table S6**. Top 10 high-cited references regarding Bevacizumab and Colorectal Cancer.

| Rank | Authors | Article Title | Source Title | Citations | Year | Document Type | DOI |
| --- | --- | --- | --- | --- | --- | --- | --- |
| 1 | Hurwitz, H et al. | Bevacizumab plus irinotecan, fluorouracil, and leucovorin for metastatic colorectal cancer | NEW ENGLAND JOURNAL OF MEDICINE | 8288 | 2004 | Article | 10.1056/NEJMoa032691 |
| 2 | Giantonio, BJ et al. | Bevacizumab in combination with oxaliplatin, fluorouracil, and leucovorin (FOLFOX4) for previously treated metastatic colorectal cancer: Results from the Eastern Cooperative Oncology Group Study E3200 | JOURNAL OF CLINICAL ONCOLOGY | 1863 | 2007 | Article | 10.1200/JCO.2006.09.6305 |
| 3 | Andre, T et al. | Pembrolizumab in Microsatellite-Instability-High Advanced Colorectal Cancer | NEW ENGLAND JOURNAL OF MEDICINE | 1230 | 2020 | Article | 10.1056/NEJMoa2017699 |
| 4 | Heinemann, V et al. | FOLFIRI plus cetuximab versus FOLFIRI plus bevacizumab as first-line treatment for patients with metastatic colorectal cancer (FIRE-3): a randomised, open-label, phase 3 trial | LANCET ONCOLOGY | 1315 | 2014 | Article | 10.1016/S1470-2045(14)70330-4 |
| 5 | Schmoll, HJ et al. | ESMO Consensus Guidelines for management of patients with colon and rectal cancer. A personalized approach to clinical decision making | ANNALS OF ONCOLOGY | 1104 | 2012 | Article | 10.1093/annonc/mds236 |
| 6 | Tol, J et al. | Chemotherapy, Bevacizumab, and Cetuximab in Metastatic Colorectal Cancer | NEW ENGLAND JOURNAL OF MEDICINE | 1085 | 2009 | Article | 10.1056/NEJMoa0808268 |
| 7 | Van Cutsem, E et al. | Addition of Aflibercept to Fluorouracil, Leucovorin, and Irinotecan Improves Survival in a Phase III Randomized Trial in Patients With Metastatic Colorectal Cancer Previously Treated With an Oxaliplatin-Based Regimen | JOURNAL OF CLINICAL ONCOLOGY | 1018 | 2012 | Article | 10.1200/JCO.2012.42.8201 |
| 8 | Bennouna, J et al. | Continuation of bevacizumab after first progression in metastatic colorectal cancer (ML18147): a randomised phase 3 trial | LANCET ONCOLOGY | 901 | 2013 | Article | 10.1016/S1470-2045(12)70477-1 |
| 9 | Lee, S et al. | Autocrine VEGF signaling is required for vascular homeostasis | CELL | 802 | 2007 | Article | 10.1016/j.cell.2007.06.054 |
| 10 | Maughan, TS et al. | Addition of cetuximab to oxaliplatin-based first-line combination chemotherapy for treatment of advanced colorectal cancer: results of the randomised phase 3 MRC COIN trial | LANCET | 790 | 2011 | Article | 10.1016/S0140-6736(11)60613-2 |
